# DIETARY RESTRICTION OF ISOLEUCINE INCREASES THE HEALTHSPAN AND LIFESPAN OF GENETICALLY HETEROGENEOUS MICE

**DOI:** 10.1093/geroni/igad104.2995

**Published:** 2023-12-21

**Authors:** Cara Green

**Affiliations:** University of Wisconsin-Madison, Madison, Wisconsin, United States

## Abstract

Low protein (LP) diets promote health and longevity in diverse species, andreducing dietary levels of the branched-chain amino acids (BCAAs) leucine, isoleucine and valine recapitulates these effects in C57BL/6J mice, promoting metabolic health in both sexes, and increases lifespan while reducing frailty in males. Each BCAA has unique metabolic effects, and we recently showed that restriction of isoleucine is both sufficient to promote metabolic health and required for the metabolic benefits of an LP diet in C57BL/6J males. Here, we tested the hypothesis that specifically restricting dietary isoleucine could promote healthy aging in adult genetically heterogenous UM-HET3 mice. We find that isoleucine restriction (IleR) improves the metabolic health of both young and old HET3 mice, promoting leanness and glycemic control in both sexes, and reprograms hepatic metabolism, blunting age-related molecular changes in males and, to a lesser extent, in females. Finally, we find that IleR reduces frailty and extends both the median and maximum lifespan of both male and female HET3 mice, but to a much greater degree in males. Our results demonstrate that restricting dietary isoleucine can increase health span and longevity in a genetically diverse population of mice and suggest that reducing dietary levels of isoleucine, or drug regimens that mimic this effect, may be a novel intervention to promote healthy aging.

